# Thermally Insulating Nanocellulose‐Based Materials

**DOI:** 10.1002/adma.202001839

**Published:** 2020-08-06

**Authors:** Varvara Apostolopoulou‐Kalkavoura, Pierre Munier, Lennart Bergström

**Affiliations:** ^1^ Department of Materials and Environmental Chemistry Stockholm University Svante Arrhenius väg 16C Stockholm 10691 Sweden

**Keywords:** aerogels, heat transfer, nanocellulose, phonon scattering, thermal insulation

## Abstract

Thermally insulating materials based on renewable nanomaterials such as nanocellulose could reduce the energy consumption and the environmental impact of the building sector. Recent reports of superinsulating cellulose nanomaterial (CNM)‐based aerogels and foams with significantly better heat transport properties than the commercially dominating materials, such as expanded polystyrene, polyurethane foams, and glass wool, have resulted in a rapidly increasing research activity. Herein, the fundamental basis of thermal conductivity of porous materials is described, and the anisotropic heat transfer properties of CNMs and films with aligned CNMs and the processing and structure of novel CNM‐based aerogels and foams with low thermal conductivities are presented and discussed. The extraordinarily low thermal conductivity of anisotropic porous architectures and multicomponent approaches are highlighted and related to the contributions of the Knudsen effect and phonon scattering.

## Graphical abstract

The recently discovered potential of cellulose nanomaterials (CNMs) as raw materials to produce foams and aerogels for thermal insulation applications is described. The fundamental heat transfer contributions in such porous materials are summarized and put into context, through a selection of works highlighting highly efficient CNM‐based isotropic and anisotropic insulation materials.

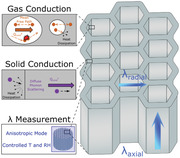

## 1 Introduction

Heat transport and thermal insulation are essential for the transport and storage of temperature sensitive products, for the thermal management of electronic devices, and to control the interior environment in buildings.^[^ ^1^, ^2^, ^3^, ^4^, ^5^, ^6^, ^7^ ^]^ The built environment stands for a substantial part of the global energy use and generates close to 30% of the global CO_2_ emissions,^[^ ^8^, ^9^, ^10^ ^]^ with space heating and cooling in buildings accounting for over 10% of the global energy consumption.^[^ ^11^ ^]^ Efficient thermal insulation based on renewable materials is an essential part of the technology change needed to mitigate climate change.^[^ ^8^, ^10^, ^12^, ^13^ ^]^ Renewable, biobased materials such as wood chips and recycled paper were extensively used for thermal insulation prior to the introduction of fossil fuel‐based foams, but their insulating performance is relatively poor and cannot compete with commercially available insulation materials such as expanded polystyrene (EPS) and polyurethane (PU) foams.^[^ ^1^ ^]^

The heat transport and thermal conductivity of low‐density foams and aerogels is commonly described as a summation of contributions from the gas conduction, solid conduction, radiation, and convection.^[^ ^14^, ^15^, ^16^, ^17^, ^18^, ^19^ ^]^ Approaches to minimize the gas thermal conductivity in a low‐density material involve the replacement of air with another gas or vacuum,^[^ ^20^, ^21^ ^]^ and the reduction of the pore size below the mean free path in air.^[^ ^3^, ^22^, ^23^, ^24^ ^]^ Heat transfer in nanostructures can be reduced by controlling the dimensions and interfaces of the materials to promote phonon scattering and maximize the interfacial thermal resistance.^[^ ^25^, ^26^ ^]^

The sheer size of the packaging and building areas makes the development of so‐called superinsulating materials, i.e., materials with thermal conductivities significantly lower than the value for air (25 mW m^−1^ K^−1^ at room temperature), crucial. Silica aerogels for instance exhibit very low thermal conductivities of 12–15 mW m^−1^ K^−1^, but they are brittle and expensive.^[^ ^19^ ^]^ With the emergence of nanosized building blocks based on renewable or widely abundant resources, there are now possibilities for the nanoscale engineering^[^ ^27^, ^28^, ^29^ ^]^ of renewable materials to generate low‐density biobased foams and aerogels that have the potential for efficient thermal insulation.^[^ ^4^, ^21^ ^]^

Nanocellulose,^[^ ^30^, ^31^ ^]^ which are rod‐like, partially crystalline cellulose nanoparticles with diameters between 3 and 50 nm and lengths from 100 nm to several micrometers, feature an attractive combination of a low density, high elastic modulus, low thermal expansion coefficient, and flexible surface chemistry. In this progress report, we will describe the heat transfer and thermal conductivity of cellulose, cellulose nanomaterials (CNMs), and wood, and give a detailed account of the composition and structural features of CNM‐based aerogels and foams with low thermal conductivities. The importance of the dimensions of the CNMs and the structure, pore size, and density of the aerogels and foams will be highlighted and related to the Knudsen effect and phonon scattering in biobased nanocomposites. CNMs are usually hygroscopic^[^ ^32^, ^33^, ^34^, ^35^, ^36^, ^37^ ^]^ and we will describe how moisture uptake modulates the heat transport and thermal conductivity of CNM‐based foams and aerogels. Finally, the ability to produce materials with aligned nanocellulose particles that display highly anisotropic heat transfer properties will be described and related to the large intrinsic anisotropy of the thermal conductivity of cellulose and the importance of phonon scattering and interfacial thermal resistance. The heat transfer properties are also related to the processing and in particular the challenges related to the removal of the solvent to generate low‐density foams and aerogels. The conclusions and outlook will elaborate on how this knowledge can be used to engineer CNM‐based aerogels, foams, and films with thermal insulation properties suitable for energy‐efficient buildings.

## 2 Thermal Conductivity: Theory and Measurements

**Figure 1:**
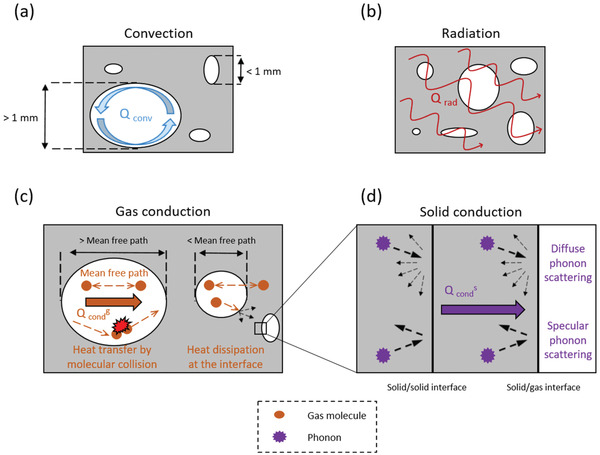
The modes of heat transport in porous materials. Heat transfer by a) convection, b) radiation, c) gas conduction, including the coupling effects at the gas–solid interface, and d) solid conduction, highlighting diffuse and specular phonon scattering at interfaces.

The conductive heat flux through a material is expressed by Fourier's Law, defined as the product of the thermal conductivity, the area, and the temperature gradient.^[^ ^7^, ^38^, ^39^ ^]^ The total heat transfer of porous materials also depends on nonconductive heat transfer processes and is commonly associated with an effective thermal conductivity, λ_eff_, that is expressed as a sum of the various modes of heat transfer (**Figure** **1**), usually classified into: solid conduction (λ^s^_cond_), gas conduction (λ^g^_cond_), convection (λ_conv_), and radiation (λ_rad_) (Equation (1))^[^ ^14^, ^15^, ^16^, ^17^, ^18^, ^19^ ^]^

(1) $$\lambda_{\text{eff}}=\lambda_{\text{cond}}^{\text{s}}\;+\;\lambda_{\text{cond}}^{\text{g}}\;+\;\lambda_{\text{conv}}\;+\;\lambda_{\text{rad}}$$

Heat transfer by convection (Figure 1a) requires that gas is transported across the temperature gradient within the material; hence the contribution from convection is negligible when the pore size of the insulating materials is sufficiently small (<1 mm).^[^ ^15^, ^17^, ^40^ ^]^ The radiation contribution (Figure 1b) is usually negligible at ambient temperature and pressure conditions, which is the main domain of interest for thermal insulation applications, but the radiation contribution can be significant at elevated temperatures (scales with *T* ^3[^ ^41^ ^]^) and under vacuum.^[^ ^17^ ^]^ However, some studies suggest that the radiative heat transfer needs to be accounted for when the effective thermal conductivity and the density of a porous material are low.^[^ ^15^, ^42^ ^]^

The gas conduction contribution, λ^g^_cond_ (Figure 1c), is the dominating contribution to the effective thermal conductivity of most low‐density foams and aerogels at ambient conditions. Because λ^g^_cond_ is based on heat transfer that proceeds through collisions of gas molecules, it can be determined experimentally by subtracting λ_eff_ measured under vacuum from λ_eff_ determined at ambient pressure conditions.^[^ ^17^, ^43^ ^]^

Gas conduction depends on the pore size and the mean free path of air molecules in the porous structures (Figure 1c) (i.e., the average distance travelled by a gas molecule between collisions),^[^ ^22^ ^]^ and is also influenced by the density and the specific surface area of the porous materials.^[^ ^23^ ^]^ The gas conduction contribution can decrease significantly if the pore size becomes smaller than the mean free path of air molecules, the so‐called Knudsen effect, which at ambient conditions (room temperature and atmospheric pressure) occurs at pore sizes below 50 nm.^[^ ^44^ ^]^

The gas–solid coupling effects^[^ ^17^, ^43^, ^45^, ^46^ ^]^ that are related to molecular collisions occurring at solid–gas interfaces, can also have a significant impact on heat transfer through the gas phase. Studies on silica aerogels^[^ ^17^, ^43^ ^]^ showed that the gas–solid coupling can be estimated from the solid particle size and the pore size.

Heat in solids is carried by electrons and phonons,^[^ ^47^ ^]^ although the role of electrons is minimal in poor electrical conductors such as cellulose.^[^ ^48^ ^]^ Phonons are energy quanta associated to atomic lattice vibrations, i.e., phonon transport is directly correlated with the strength and density of atomic bonding.^[^ ^25^, ^48^, ^49^, ^50^ ^]^ Covalent bonds favor heat transfer while phonons propagate less effectively through weaker bonds such as hydrogen and van der Waals bonds.^[^ ^25^, ^37^, ^39^, ^48^, ^51^, ^52^ ^]^ The solid contribution to the thermal conductivity can be reduced by phonon scattering at interfaces (Figure 1d), often referred to as the interfacial thermal resistance.^[^ ^27^, ^40^, ^53^, ^54^ ^]^ Hence, nanostructured and nanoporous materials are expected to exhibit a low solid conduction contribution due to phonon scattering at the solid–solid and solid–gas interfaces.^[^ ^55^, ^56^, ^57^ ^]^

Approaches to describe heat transfer at solid–solid interfaces in porous materials include estimates of the Kapitza resistance,^[^ ^29^, ^54^, ^58^, ^59^ ^]^ geometrical or density‐related approaches,^[^ ^14^, ^15^, ^18^, ^40^, ^60^ ^]^ or estimates based on the sound velocity^[^ ^43^, ^61^ ^]^ or the phonon mean free path.^[^ ^7^, ^17^, ^41^, ^55^, ^56^, ^62^ ^]^ Such estimates are rare for cellulose‐based materials but Coquard and Baillis performed Monte Carlo simulations to estimate λ^s^_cond_ of a porous insulation material based on a cellulosic matrix in vacuum.^[^ ^41^ ^]^ They found that the diffuse scattering of phonons increases with a reduction of the pore size below the mean free path of phonons. Engineering materials with a high density of thin interfaces increases the possibility for diffuse scattering, which favors energy dissipation and results in a reduction of the thermal conductivity.^[^ ^29^, ^55^ ^]^ The importance of phonon scattering was demonstrated in a recent study on a graphene aerogel, which exhibited a thermal conductivity of only 5–6 mW m^−1^ K^−1^ in vacuum at room temperature.^[^ ^57^ ^]^

Replacement of air with water through moisture uptake of hygroscopic materials, such as wood, cellulose, and CNMs,^[^ ^32^, ^33^, ^34^, ^35^, ^36^, ^37^ ^]^ usually results in an increase of the heat conduction because water has a higher thermal conductivity than air. Relative humidity (RH) and temperature control the moisture uptake and it is thus important to determine how the thermal conductivity of cellulose‐based materials depends on the RH and/or the water content in the material. Künzel investigated how the thermal conductivity of wood and other hydrophilic building materials depends on the moisture content and proposed a model that predicts that the thermal conductivity increases linearly with the volumetric moisture content.^[^ ^63^, ^64^ ^]^ Apostolopoulou‐Kalkavoura et al. showed that the Künzel model substantially underestimates the effect of moisture uptake on the thermal conductivity of highly hygroscopic foams based on cellulose nanofibers (CNFs) and nonionic polyoxamers, and presented a modified model where the wet effective thermal conductivities increase linearly with the moisture content by mass.^[^ ^65^ ^]^ Ochs et al. proposed an engineering model for moisture‐containing materials where the thermal conductivity is estimated by a summation either in parallel or in series of contributions from the solid, the gas, and diffusion due to evaporation in the pores and the liquid water.^[^ ^66^ ^]^ Unfortunately, it is rare that studies on the heat transfer properties of CNMs report at what RH the thermal conductivity has been determined and it is very unusual that the RH‐dependence of the thermal conductivity has been measured.

The thermal conductivity can be measured by either steady state or transient techniques. The steady state techniques measure the heat flow across a sample of a known thickness that is held at a sufficiently large steady state temperature gradient.^[^ ^7^, ^67^, ^68^, ^69^ ^]^ Two examples of steady state techniques are the guarded hot plate^[^ ^43^, ^46^, ^63^, ^70^ ^]^ and the heat flow meter apparatus.^[^ ^40^, ^71^, ^72^, ^73^, ^74^ ^]^ The transient techniques determine the energy dissipation through a sample that has been subjected to a heat pulse.^[^ ^7^, ^67^, ^69^ ^]^ The hot wire method^[^ ^18^, ^23^, ^41^, ^45^, ^60^, ^68^, ^75^, ^76^ ^]^ and the hot strip method,^[^ ^62^, ^77^, ^78^, ^79^ ^]^ in which a linear and a planar heat source, respectively, are embedded in the sample, are commonly used transient techniques. The transient plane source (TPS), or hot disk,^[^ ^59^, ^65^, ^80^, ^81^, ^82^, ^83^, ^84^, ^85^, ^86^ ^]^ is a recent development of the hot wire and hot strip techniques,^[^ ^67^, ^87^ ^]^ where a strip or disk is sandwiched between two identical samples.

The steady state techniques are suitable to estimate the thermal conductivity of relatively large samples and are commonly used to determine the thermal conductivity of insulating materials. The transient techniques are commonly used to determine the thermal conductivity of smaller samples. Most methods require that there is good contact between the thermocouples and the sample, although contact issues between sample and sensor can sometimes be corrected for in some of the transient techniques.

The laser flash^[^ ^26^, ^46^, ^88^, ^89^ ^]^ is a noncontact and noninvasive transient technique that measures the thermal diffusivity.^[^ ^67^, ^68^, ^69^ ^]^ The thermal conductivity of thin films can also be determined by transient methods, e.g., the 3ω technique^[^ ^47^, ^90^, ^91^, ^92^ ^]^ or the time‐domain thermoreflectance (TDTR).^[^ ^93^, ^94^ ^]^

## 3 Thermal Conductivity of Cellulose, Wood, and CNM‐Based Films

**Figure 2:**
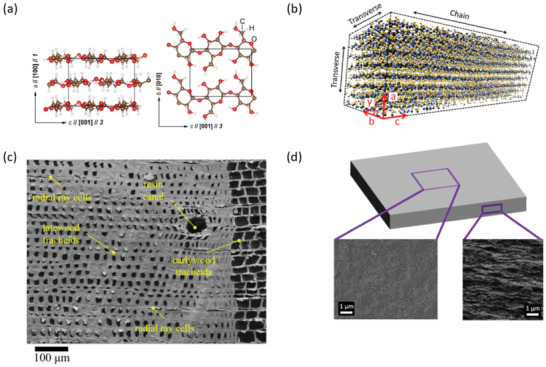
Crystalline structure of cellulose and morphology of wood‐ and nanocellulose‐based films. a) Schematic views of cellulose Iβ crystal units. b) Schematic representation of a cellulose nanocrystal (CNC) composed of aligned cellulose chains. c) Visualization of the porous structure of wood. d) Schematic of a CNF film obtained by vacuum filtration, with SEM images corresponding to in‐plane (left) and thickness (right) views. a) Adapted with permission.^[^ ^39^ ^]^ Copyright 2014, IOP Publishing. b) Adapted with permission.^[^ ^48^ ^]^ Copyright 2014, American Chemical Society. c) Adapted with permission.^[^ ^107^ ^]^ Copyright 2015, Springer Nature. d) Adapted with permission.^[^ ^108^ ^]^ Copyright 2018, American Chemical Society.

Cellulose exists in two different crystalline forms Iα and Iβ. Cellulose Iα is mainly found in green algae and bacteria while cellulose Iβ is the most common form of cellulose existing in wood, tunicates, and cotton.^[^ ^95^, ^96^, ^97^, ^98^ ^]^ Cellulose Iα has a one‐chain triclinic unit cell and cellulose Iβ (**Figure** **2**a) has a two‐chain monoclinic unit cell.^[^ ^31^, ^95^, ^99^, ^100^ ^]^ Both cellulose crystal structures are characterized by covalent bonds along the *c*‐axis and weaker van der Waals and hydrogen bonds along the *a* and *b* directions.^[^ ^95^, ^98^, ^101^, ^102^ ^]^

Estimating the thermal conductivity of cellulose Iβ crystals (**Table** **1**) by modeling showed that its heat transfer properties are anisotropic, with values of about 900 mW m^−1^ K^−1^ along the “axial” *c*‐axis (λ_a_), and 240 and 520 mW m^−1^ K^−1^ along the “radial” *a* and *b* (λ_r_) axes, respectively, at 298 K.^[^ ^39^ ^]^ Estimates of the thermal conductivity in vacuum of a single cellulose nanocrystal (CNC), modeled as a parallelepipedic bundle of aligned cellulose chains by molecular dynamics simulations (Figure 2b), resulted in an axial thermal conductivity of 5700 mW m^−1^ K^−1^,^[^ ^48^ ^]^ which is much higher than the estimate for the cellulose Iβ crystals,^[^ ^39^ ^]^ and 7.9 times higher than the estimated radial thermal conductivity (720 mW m^−1^ K^−1^).^[^ ^48^ ^]^

**Table 1:** Thermal conductivity of cellulose‐, wood‐, and CNM‐based films

Refs.	Material	Density [kg m^−3^]	λ_a_ [mW m^−1^ K^−1^]	λ_r_ [mW m^−1^ K^−1^]	λ_a_/λ_r_	*T* [K]	RH [%]
^[^ ^39^ ^]^	Cellulose Iβ	1500–1600	900	240^a)^/500^b)^	3.8/1.8	298	N/A
^[^ ^48^ ^]^	CNC	1500–1600	5700^c)^	720^c)^	7.9	300	–
^[^ ^103^ ^]^	Partly crystalline cellulose in wood	1500–1600	1040	260	4.0	293	N/A
^[^ ^104^ ^]^	Wood fibers	1500–1600	766	430	1.8	293	N/A
^[^ ^104^ ^]^	Birch	680	323	214	1.5	294	30
^[^ ^80^ ^]^	Oak	753	270	160	1.7	293	30
^[^ ^48^ ^]^	Shear‐oriented CNC films	N/A	530^c)^	220^c)^	2.4	300	–
^[^ ^105^ ^]^	TNW^d)^ nanopaper	1090	2470	290	8.5	298	N/A
^[^ ^105^ ^]^	TOSNF^e)^ nanopaper	1100	635	360	1.8	298	N/A

a) *a*‐axis of the unit cell

b) *b*‐axis of the unit cell

c) Under vacuum

d) Tunicate nanowhiskers

e) TEMPO‐oxidized Sugi cellulose nanofiber.

Eitelberger and Hofstetter estimated the thermal conductivity of wood fibers or, as it was expressed in the study, “partly crystalline cellulose as it is found in wood,” by combining models for stretched polymers with the thermal expansion coefficients and the thermal conductivity value for glassy glucose.^[^ ^103^ ^]^ The thermal conductivity of wood fibers was found to be anisotropic with an axial thermal conductivity (along the chain) of 1040 mW m^−1^ K^−1^ and a radial thermal conductivity of 260 mW m^−1^ K^−1^. In an early estimate of the thermal conductivity of wood fibers, Suleiman et al. reported values of 766 mW m^−1^ K^−1^ along the fibers and 430 mW m^−1^ K^−1^ perpendicularly to them.^[^ ^104^ ^]^ It should be noted that the majority of the estimates of thermal conductivity assume the bulk density of cellulose to be 1500 kg m^−3^; however, it should be pointed out that Daicho et al.^[^ ^106^ ^]^ recently demonstrated that the true density of fibrillary crystallites of cellulose is closer to 1600 kg m^−3^.

The thermal conductivity of wood (Figure 2c) is anisotropic, e.g., the thermal conductivity of birch is 1.5 times higher in the longitudinal (λ_a_) direction, i.e., along the direction of the lumen (323 mW m^−1^ K^−1^ at 294 K), than in the transverse (λ_r_) direction (214 mW m^−1^ K^−1^ at 294 K).^[^ ^104^ ^]^ The anisotropy in the thermal conductivity of wood is lower than for cellulose crystals, which is related to the hierarchical structure of wood and the fact that wood contains other biopolymers that do not possess the same intrinsic anisotropic heat transfer properties as cellulose.

Films consisting of aligned CNC particles also exhibit an anisotropic thermal conductivity. Diaz et al. obtained an anisotropy ratio of 2.4 with a thermal conductivity of 530 mW m^−1^ K^−1^ along the fibers’ (λ_a_), and 220 mW m^−1^ K^−1^ perpendicularly to the fibers’ (λ_r_) directions.^[^ ^48^ ^]^ The anisotropic thermal conductivity of CNM‐based materials is affected by the composition, the crystallinity, or the crystallite size, and of course the degree of alignment of nanocellulose in the materials. Nanocellulose films (Figure 2d) that are produced using thin and highly crystalline nanocellulose particles or fibrils result in a low thermal conductivity with a large interfacial thermal resistance caused by the high density of internal interfaces.^[^ ^29^, ^55^, ^105^ ^]^ The interfacial resistance could potentially be increased even further, resulting in ultralow thermal conductivity, by mixing CNMs and other components of different shapes and aspect ratios. Uetani et al. prepared CNM films based on TEMPO‐oxidized Sugi cellulose nanofiber (TOSNF) with a thermal anisotropy ratio of 1.8, and nanopapers based on aligned tunicate nanowhiskers (TNWs) with a thermal anisotropy ratio of 8.5.^[^ ^105^ ^]^

## 4 Isotropic CNM/Cellulose‐Based Aerogels and Foams

Aerogels with low density and pores smaller than the mean free path of air can display thermal conductivities significantly lower than the value for air. The prime example reported is mesoporous silica aerogels, with densities typically between 80 and 200 kg m^−3[^ ^19^, ^74^, ^109^, ^110^ ^]^ with reports of densities as low as 1.29 kg m^−3^,^[^ ^72^ ^]^ which can display a λ as low as 12–15^[^ ^19^, ^74^, ^110^ ^]^ mW m^−1^ K^−1^ at ambient conditions.

Silica aerogels consist of noncrystalline silica clusters that form a 3D gel with pores smaller than 50 nm.^[^ ^110^ ^]^ Hence, the pore structure of silica aerogels is isotropic and there is no preferred crystallographic orientation because the solid material is amorphous. The thermal conductivity is the same in all the directions and it is thus sufficient to characterize the heat transfer properties for an isotropic material with a single value for the thermal conductivity. CNM‐based aerogels and foams with isotropic pores and no preferred orientation of the crystalline fibrillary nanoparticles can also be characterized as a material with isotropic heat transport properties. Isotropic CNM‐based aerogels and foams can be produced by several different routes. The removal of the solvent is a time‐consuming and energy‐demanding step that often determines the final structure and pore‐size distribution of the low‐density aerogels and foams. Supercritical drying (SCD) and freeze‐drying (FD) are commonly used to generate materials with minimal shrinkage and structural change while evaporation of the solvent, usually water, at ambient conditions or in an oven, is energy‐effective and scalable but may result in shrinkage and pore collapse due to the drying stresses.^[^ ^3^, ^19^, ^111^ ^]^ The terms “aerogel” and “foam” are used somewhat arbitrarily in the literature. The IUPAC definition of an aerogel (“gel comprising a microporous solid in which the dispersed phase is a gas”)^[^ ^112^ ^]^ suggests that the pore size should be smaller than 2 nm but the most well‐known aerogels, silica aerogels, typically have pore sizes around 10–50 nm.^[^ ^110^ ^]^ Lavoine and Bergström suggested that lightweight porous materials based on nanocellulose should be called aerogels if the pore size is less than 50 nm and foams if the pore size is larger than 50 nm.^[^ ^111^ ^]^ For simplicity, this progress report will normally adopt the terminology used in each publication and in certain cases add explanatory notes.

### 4.1 Supercritically Dried CNM/Cellulose‐Based Aerogels and Foams

SCD is based on the removal of a fluid at supercritical conditions to inhibit the formation of a liquid/vapor interface and capillary pressure‐induced stresses during the solvent removal.^[^ ^3^, ^19^, ^111^ ^]^ SCD often relies on the replacement of water with a fluid that can be supercritically dried at lower pressures and temperatures, such as carbon dioxide.

**Figure 3:**
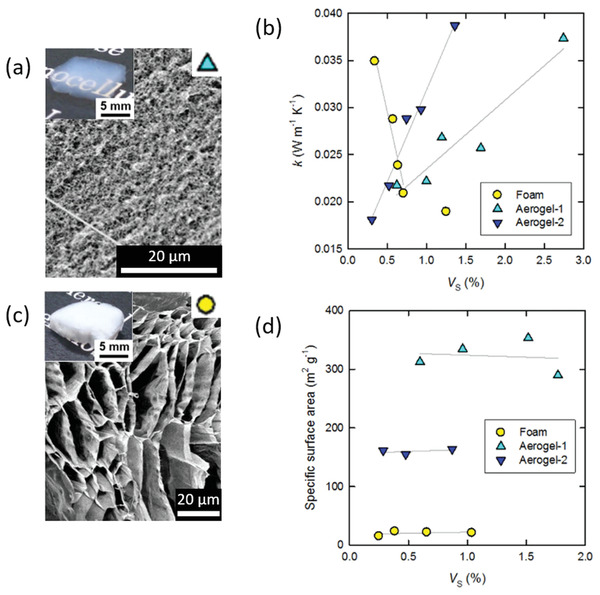
CNF‐based supercritically dried aerogels and freeze‐dried foams. a) SEM and optical microscopy images of a supercritically dried CNF aerogel. b) Thermal conductivity as a function of the solid volume fraction in the dispersions for ice‐templated/freeze‐dried foams and two types of supercritically dried aerogels (prepared by various gelation and solvent exchange protocols). c) SEM and optical microscopy images of an ice‐templated/freeze‐dried CNF foam. d) Specific surface area as a function of solid volume fraction for the same materials as in (b). Adapted with permission.^[^ ^114^ ^]^ Copyright 2016, Springer Nature.

Isotropic nanocellulose aerogels have been produced by SCD of cellulose nanofibril gels using different gelation protocols and solvent exchange procedures.^[^ ^113^, ^114^ ^]^ The thermal conductivity depended on the density of the aerogels and the lowest thermal conductivity of 18 mW m^−1^ K^−1^ was obtained for aerogels based on very thin CNFs (thickness around 3 nm) and pore sizes around 30 nm and densities of 17 kg m^−3^ (**Figure** **3**a).^[^ ^113^ ^]^ The thermal conductivity increased up to 38 mW m^−1^ K^−1^ with an increase in the density to 40 kg m^−3^ but the porosity (98.1–99.7%) and the surface area (500–600 m^2^ g^−1^) remained high through the whole density range tested (Figure 3b). It is interesting to note that the supercritically dried CNF aerogels with the lowest thermal conductivity (18 mW m^−1^ K^−1^) are superinsulating with a thermal conductivity almost as low as supercritically dried silica aerogels (12–15 mW m^−1^ K^−1^).^[^ ^19^ ^]^ This shows that the relatively small pore size and probably also the interfacial thermal resistance of the fibrils in the aerogel/foam walls contribute to reduce the thermal conductivity and suggest that the diameter and aspect ratio of celluloses and CNMs can have a significant influence on the heat transfer properties of the isotropic aerogels and foams.

Aerogels with densities of 9–137 kg m^−3^, porosities of 91–99%, and pore sizes ranging from 10–100 nm up to 1 µm, which were prepared by SCD of microcrystalline cellulose with a diameter of 10–20 nm, displayed thermal conductivities of 40–75 mW m^−1^ K^−1^, measured at RT and unspecified RH.^[^ ^85^ ^]^ The aerogel with the lowest density (9 kg m^−3^) displayed a thermal conductivity as high as 40 mW m^−1^ K^−1^, which suggests that the pore sizes are too large to result in a reduction of the gas conduction contribution by Knudsen effects and that the interfacial thermal resistance is insignificant due to the relatively large dimensions (diameter) of the microcrystalline cellulose.

In addition to isotropic aerogels based on only nanocellulose, there are examples of CNM‐based composite^[^ ^76^, ^122^, ^123^, ^124^ ^]^ or hybrid^[^ ^75^ ^]^ aerogels that display low thermal conductivities. SCD of cellulose acetate‐isocyanate sols resulted in aerogels with a relatively high density of 250 kg m^−3^, large surface area (250 m^2^ g^−1^), small pore size (25 nm), and a thermal conductivity of 29 mW m^−1^ K^−1^ at RT and RH.^[^ ^75^ ^]^ It should be noted that these aerogels do not contain any crystalline cellulose and it is thus reasonable to compare their insulating performance with polymer‐based aerogels or even commercially available insulating materials such as polystyrene, with a density of 25–45 kg m^−3^ and a λ of 35–45 mW m^−1^ K^−1^,^[^ ^125^ ^]^ or rigid polyurethane foams with a density of 30 kg m^−3^ and a λ of 25 mW m^−1^ K^−1^.^[^ ^126^ ^]^

### 4.2 Freeze‐Dried CNM/Cellulose‐Based Aerogels and Foams

The preparation of freeze‐dried aerogels and foams involves a rapid solidification of the dispersions, typically by immersion in liquid nitrogen, followed by removal of the frozen solvent (normally water) by sublimation at conditions below the solvent's triple point to prevent melting and the formation of a liquid/vapor interface.^[^ ^3^, ^111^ ^]^ FD aerogels of very low density (1–8 kg m^−3^), prepared from TEMPO‐oxidized cellulose nanofiber (TCNF) and CNF ultrasonicated at a high intensity, exhibited thermal conductivities below 16 mW m^−1^ K^−1^, which is one of the lowest thermal conductivities reported for FD CNM aerogels but the measurements were unfortunately performed at an unspecified *T* and RH.^[^ ^115^ ^]^ A direct comparison between FD and SCD is offered by Sakai et al., where aerogels/foams based on the same CNF materials were prepared by both SCD and FD (Figure 3c).^[^ ^114^ ^]^ The FD foams with densities between 4 and 20 kg m^−3^ featured pores with sizes of several micrometers with thin but dense walls consisting of assembled CNF, displayed a thermal conductivity of 19–35 mW m^−1^ K^−1^ (measured at 296 K and 50% RH) that actually decreased with increasing density (Figure 3b). The SCD aerogels (Figure 3a) featured much smaller pores in a nanofibrous network‐like solid skeleton and a surface area that is 8 times higher than the FD foams (Figure 3d), and displayed thermal conductivities of 18–40 mW m^−1^ K^−1^, which increased with the density of the aerogels (Figure 3b). The decrease in thermal conductivity with increasing density for the FD foams was related to a reduction of the gas conduction by the authors, but it is also possible that the introduction of more fibril–fibril interfaces in the pore walls caused the interfacial thermal resistance to increase.

Other studies have also observed that the thermal conductivity of FD aerogels and foams can decrease with increasing density. Jiménez‐Saelices et al. found that the thermal conductivity of FD CNF aerogels prepared by freezing Pickering emulsions of hexadecane in water^[^ ^77^ ^]^ displayed a parabolic behavior with increasing density, where the λ decreased to a minimum (18 mW m^−1^ K^−1^ for the aerogel density of 20 kg m^−3^) and then increased for densities up to 30 kg m^−3^. Seantier et al. showed that the addition of 10 wt% of CNM was able to reduce the thermal conductivity of aerogels made of bleached cellulose fibers; from 28 mW m^−1^ K^−1^ for cellulose fiber aerogels to 23 mW m^−1^ K^−1^ with the addition of 10 wt% CNF (with 20 ± 4 nm diameter and 300 ± 20 nm length) and 25 mW m^−1^ K^−1^ with the addition of 10 wt% CNC (**Table** **2**).^[^ ^119^ ^]^ The pore size was substantially reduced by the addition of CNF or CNC and the all‐cellulose multiscale composites also displayed a reduction of the thermal conductivity as the density of the aerogels was increased by controlled compression. Hence, this work confirms that the thermal conductivity of isotropic FD aerogels and foams can decrease with increasing density, but the cause of the reduction in thermal conductivity is unclear. An increase in the density of an aerogel or foam will always result in an increase in the solid conduction contribution so the observed decrease of the effective thermal conductivity suggests that other contributions are significantly reduced as the density is increased. The preparation of foams and aerogels by FD usually generates smaller pore sizes and thicker foam walls if the CNM concentration in the dispersion to be frozen is higher, which can result in a reduction of the gas conduction contribution and an increase in the interfacial thermal resistance with increasing density, respectively. It has also been suggested that the radiative heat transfer could decrease with increasing density.^[^ ^77^ ^]^

**Table 2:** Thermal conductivity of isotropic CNM/cellulose‐based aerogels and foams

Refs.	Drying	Cellulose type	Cellulose	Other components	λ^c)^	RH^d)^	ρ^e)^	*Π* ^f)^	Pore size
			*d* ^a)^/*L* ^b)^		[mW m^−1^ K^−1^]	[%]	[kg m^−3^]	[%]	
^[^ ^113^, ^114^ ^]^	SCD^g)^	TCNF^h)^	3 nm/430 nm	–	18–38	50	4–40	98.1–99.7	30 nm
^[^ ^71^ ^]^	SCD	Regenerated cellulose	N/A/N/A	–	33	N/A	20–30	>98	nm to µm
^[^ ^85^ ^]^	SCD	Microcrystalline cellulose	10–20 nm/N/A	–	40–75	N/A	9–137	91–99	10 nm to 1 µm
^[^ ^75^ ^]^	SCD	Cellulose acetate	N/A/N/A	Isocyanate	29	N/A	250	47	25 nm
^[^ ^73^, ^74^ ^]^	SCD	Short cellulosic fibers	14 µm/2 mm	Silica	15	N/A	100–130	>90	nm
^[^ ^114^ ^]^	FD^i)^	TCNF	3 nm	–	18–40	50	4–20	97.3–99.7	30 nm, µm
^[^ ^78^ ^]^	(Spray) FD	TCNF	3.9 nm/450 nm	–	18–21	50	12–33	98–99	nm to µm
^[^ ^78^ ^]^	FD	TCNF	3.9 nm/450 nm	–	24–28	50	12–33	98–99	nm to µm
^[^ ^115^ ^]^	FD	TCNF	2–9 nm/50–400 nm	–	<16	N/A	1–8	>99	nm to µm
^[^ ^116^ ^]^	FD	CNF	N/A/N/A	–	39.6	N/A			
^[^ ^117^ ^]^	FD	BC^j)^	N/A/N/A	–	29.5	N/A	7	99.6	N/A
^[^ ^77^ ^]^	FD	TCNF	4 nm/450 nm	Hexadecane	18–30	50	12–30	98–99	nm to µm
^[^ ^118^ ^]^	FD	TCNF	N/A/N/A	CA^k)^	70	N/A	200	85	nm to µm
^[^ ^119^ ^]^	FD	Bleached cellulose fibers, CNMs	10 µm/700 µm for cellulose 7–40 nm/300–2000 nm for CNMs	–	23–28	N/A	15–160	N/A	nm to µm
^[^ ^120^ ^]^	FD	CNF and cellulose microfibers	25 µm/500 µm for cellulose 20 nm/300 nm for CNMs	Nanozeolites	18–31	N/A	N/A	N/A	nm
^[^ ^70^ ^]^	FD	CNF and silylated‐CNF	10–100 nm/500–10 000 nm	Silica	13.8–33.9	N/A	7–146	94–99.4	nm to µm
^[^ ^83^ ^]^	FD	CNF	20 nm/N/A	MOF	41–55	7–80	0.2–3	>99	nm to µm
^[^ ^121^ ^]^	FD	Recycled cellulose fibers	8 µm/N/A	Methyltrimethoxysilane	29–32	N/A	40	94.8	40–200 µm
^[^ ^73^, ^74^ ^]^	AD^l)^	Short natural, recycled cellulosic fibers	10–14 µm/2–12 mm	Silica	16–21	N/A	108–130	>90	nm
^[^ ^65^ ^]^	OV^m)^	TCNF	2.4 nm/0.1–1.5 µm	P123, CaCO_3_	46–86	7–80	11.9	99	145 µm

a) *d*: diameter

b) *L*: length

c) λ: thermal conductivity

d) RH: relative humidity

e) ρ: density

f) *Π*: porosity

g) SCD: supercritical drying

h) TCNF: TEMPO‐oxidized cellulose nanofibers

i) FD: Freeze‐drying

j) Bacterial cellulose

k) Cellulose acetate

l) Ambient drying

m) Oven drying.

**Figure 4:**
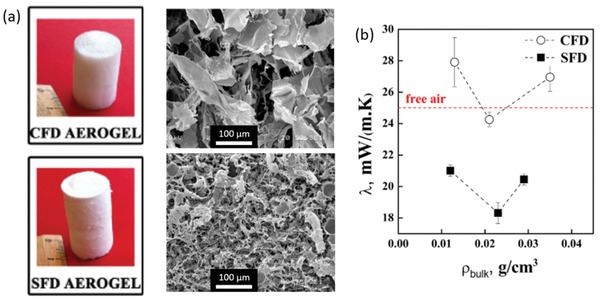
Influence of freezing techniques on the structure and properties of freeze‐dried aerogels. a) Macroscopic and SEM views of a CNF aerogel prepared by conventional freeze‐drying (top) and spray freeze‐drying (bottom). b) Thermal conductivity as a function of bulk density for the corresponding aerogels. Adapted with permission.^[^ ^78^ ^]^ Copyright 2017, Elsevier.

Thermal conductivities as low as 18 mW m^−1^ K^−1^ were obtained for spray freeze‐dried (SFD) CNF aerogels with a density of 23 kg m^−3^ at 295 K and 50% RH.^[^ ^78^ ^]^ In this work, the authors compared conventional FD, during which the CNF dispersion is frozen at 193 K for 24 h, and SFD, during which the CNF dispersion is sprayed layer by layer onto moulds that have been cooled to 193 K (**Figure** **4**a). The SFD CNF aerogels exhibited a lower thermal conductivity (18 mW m^−1^ K^−1^) (Figure 4b) because they formed a 3D network consisting of very small pores (10–100 nm) due to the faster freezing, compared to the conventionally and more slowly frozen foams (24 mW m^−1^ K^−1^), which displayed a macroporous and 2D sheet‐like wall structure. The addition of a TCNF aerogel in a hollow fiber with a diameter of 0.85 mm made from cellulose acetate results in reducing the thermal conductivity from 100 mW m^−1^ K^−1^, for the neat hollow fiber, to 70 mW m^−1^ K^−1^ for the multiscale material, at 299 K but undefined RH.^[^ ^118^ ^]^ Freeze dried bacterial cellulose (BC) aerogels with a density of 7 kg m^−3^ exhibited a thermal conductivity of 29.5 mW m^−1^ K^−1^.^[^ ^117^ ^]^ The heat transfer properties of FD foams and aerogels based on CNF prepared from cotton, wood, bamboo, and rice straw displayed thermal conductivity values, at 298 K and undefined RH, which ranged from 39.6 mW m^−1^ K^−1^ at a density of 7.51 kg m^−3^ (wood) to 45.5 mW m^−1^ K^−1^ at a density of 5.92 kg m^−3^ (rice straw).^[^ ^116^ ^]^ Interestingly, only the cotton CNF aerogel (4.97 kg m^−3^), which exhibited the highest degree of crystallinity (87%), did not degrade at 473 K and exhibited a thermal conductivity of 54.5 mW m^−1^ K^−1^ at these elevated temperatures. The thermal conductivities of these FD aerogels were relatively high despite a low density, which indicates that the defibrillation may have been unsuccessful but no information on the fibrils’ dimensions was reported.

**Figure 5:**
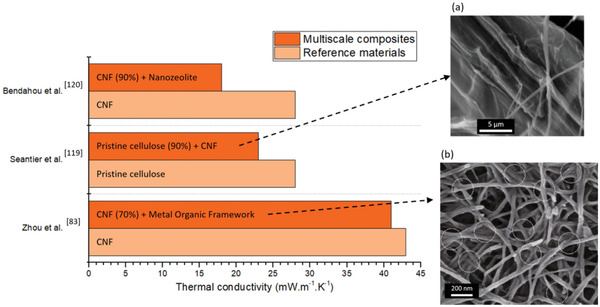
Heat transfer in multiscale cellulose/CNM foams and aerogels. Graphical summary comparing the thermal conductivity of multiscale foams and aerogels based on cellulosic materials with the corresponding reference materials. The SEM images (a) and (b) refer to the materials to which they are linked with dashed arrows. Experimental values from refs. ^[^ ^83^, ^119^, ^120^ ^]^. a) Adapted with permission.^[^ ^119^ ^]^ Copyright 2016, Elsevier. b) Adapted under the terms and conditions of the CC BY 4.0 license.^[^ ^83^ ^]^ Copyright 2020, The Authors, published by Springer Nature.

Multiscale and multimaterials approaches where components of varying sizes and aspect ratios are used to prepare aerogels and foams, with hierarchical porosities and heterogeneous interfaces, have resulted in materials with low thermal conductivities (**Figure** **5**). Composites based on celluloses with relatively large dimensions usually exhibit thermal conductivities higher than the value for air.^[^ ^83^, ^117^, ^127^, ^128^, ^129^, ^130^ ^]^ FD foams of a mixture of microcrystalline cellulose, montmorillonite, and surfactant Tween‐80 exhibited a thermal conductivity of 32.6 mW m^−1^ K^−1^.^[^ ^130^ ^]^ CNF–metal organic framework aerogels exhibited a thermal conductivity of 41 mW m^−1^ K^−1^ (at 295 K and 7% RH) that was slightly lower compared to the CNF‐only aerogel (43 mW m^−1^ K^−1^).^[^ ^83^ ^]^ The addition of 10 wt% of zeolites to CNF aerogels^[^ ^120^ ^]^ resulted in a thermal conductivity of 18 mW m^−1^ K^−1^ (at unspecified measurement conditions), and adding zeolites to significantly thicker cellulose microfibers (CMF) to generate CMF–zeolite aerogels resulted in a much higher thermal conductivity of 31 mW m^−1^ K^−1^ compared to the CNF–zeolite aerogels. The main difference between the CNF–zeolite and the CMF–zeolite aerogels is the pore size distribution, where the former aerogels are dominated by mesopores, and the latter aerogels display significantly larger macropores.

Hybrid silylated silica/CNF aerogels with a CNF content of less than 10 wt% and a density of 130 kg m^−3^ displayed a thermal conductivity as low as 13.8 mW m^−1^ K^−1^, which was only 1–2 mW m^−1^ K^−1^ larger than the silica‐only aerogel.^[^ ^70^ ^]^ It is interesting to point out that the nonsilylated silica/CNF aerogel with a density of 122 kg m^−3^ had a thermal conductivity of 17.6 mW m^−1^ K^−1^, which is 30% larger than the silylated hybrid. The reference CNF aerogel (7.4 kg m^−3^) exhibited a much higher thermal conductivity of 33.9 mW m^−1^ K^−1^, which was slightly reduced after silylation (31.3 mW m^−1^ K^−1^, at a density of 9.8 kg m^−3^). The lower thermal conductivity of the silylated compared to the nonsilylated hybrid and CNF aerogels can be related to the increased hydrophobicity of the silylated hybrids and their reduced water uptake.^[^ ^70^ ^]^ Indeed, the CNF before silylation exhibited a moisture uptake of 27% while, after the silylation, the water uptake was only 14.5% at 95% RH. The effect of hydrophobization to control the water uptake was also studied by Nguyen et al. who prepared cellulose aerogels with a density of 40 kg m^−3^ based on recycled cellulose fibers with a width of 8 µm.^[^ ^121^ ^]^ The thermal conductivity of the unmodified aerogels was 32 mW m^−1^ K^−1^ (at RT and unspecified RH), which was decreased to 29 mW m^−1^ K^−1^ when the aerogel was hydrophobized with methyltrimethoxysilane. It is clear that, while it appears that the reduction of the pore sizes and the increase in the number of heterogeneous particle–particle interfaces can contribute to reduce both the gas and solid conduction in composite multiscale aerogels,^[^ ^70^, ^119^, ^120^ ^]^ there is a lack of studies that have thoroughly characterized the structural features and related them to the heat transfer properties.

### 4.3 Oven‐ or Ambient‐Dried CNM/Cellulose‐Based Foams and Aerogels

Ambient or oven drying of wet CNM/cellulose‐based foams or aerogels could offer a cost‐effective way for large‐scale production of CNM‐based thermally insulating materials but this requires that the drying‐induced shrinkage that often results in a strongly distorted or even collapsed porous structure can be mitigated. The effect of the capillary stresses^[^ ^111^ ^]^ during solvent removal can be reduced by, e.g., reinforcement of the nanofibrillar network by crosslinking, and/or by reducing the contact angle of water by hydrophobization.^[^ ^3^, ^131^, ^132^ ^]^ Cellulose–silica aerogels with relatively small shrinkage (5–7%) could be prepared by ambient drying (AD) where the composite fibrillar network provided the gel with a sufficient strength to withstand the drying stresses.^[^ ^73^, ^74^ ^]^ The thermal conductivities of the AD cellulose–silica aerogels with a cellulose content of 9–27 wt% were only 1–2 mW m^−1^ K^−1^ higher than the equivalent hybrid aerogels prepared by SCD. Cellulose–silica aerogels based on short natural and recycled cellulosic fibers displayed thermal conductivities of 17 and 20–21 mW m^−1^ K^−1^, respectively,^[^ ^73^ ^]^ while TENCEL fiber–silica AD aerogels displayed a thermal conductivity of 16 mW m^−1^ K^−1^.^[^ ^74^ ^]^

Oven dried CNF/polyoxamer‐based foams (11.9 kg m^−3^) were prepared by crosslinking the CNF in the lamella of the wet polyoxamer‐stabilized CNF foams by pH‐triggered release of calcium.^[^ ^65^ ^]^ The thermal conductivity of the isotropic CNF/polyoxamer‐based foams was 46 mW m^−1^ K^−1^ at 295 K and 7% RH, and increased slightly to 48 mW m^−1^ K^−1^ with an increase in temperature to 313 K and 2% RH, where the RH in both cases corresponded to 1.18 g H_2_O m^−3^. The CNF/polyoxamer‐based foams were highly hygroscopic and the thermal conductivity increased by about 87% when the RH increased from 7% to 80% RH at 295 K. The importance of moisture uptake on the heat transfer of CNM‐based aerogels and foams cannot be understated and it is thus recommended that the thermal insulation performance of all CNM–cellulose‐based materials should be tested over a broad range of RH to be representative.

## 5 Anisotropic CNM/Cellulose‐Based Foams

CNM‐based foams and films with anisotropic structures and aligned CNMs can display highly anisotropic heat transfer properties. The anisotropic thermal conductivity is related to the intrinsic heat transfer anisotropy of CNMs, as discussed in Section 3 (Table 1), and the highly anisotropic shape of the rod‐like CNMs, which results in a highly anisotropic distribution and density of internal interfaces in materials with aligned CNMs. It is possible that directional heat transport and thermal insulation could be of interest in packaging and also to control the temperature and interior environment in buildings.

**Figure 6:**
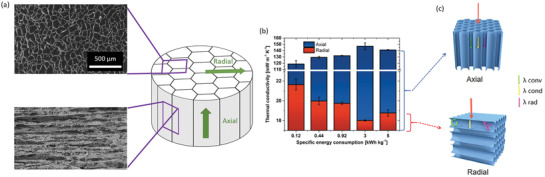
Ice‐templated and freeze‐dried CNM‐based porous structures and their thermal conductivity. a) Illustration supported with SEM images of the structure of an ice‐templated foam; the SEM images show the cross‐section or radial direction (left) and along the macropores, i.e., the axial direction (right). b) The axial and radial thermal conductivities of ice‐templated CNF foams as a function of energy consumption during defibrillation, which relates to the degree of fibrillation. c) Visualization of the anisotropic heat transfer in columnar porous structures produced by unidirectional freezing. a,b) Adapted with permission.^[^ ^82^ ^]^ Copyright 2018, American Chemical Society. c) Adapted with permission.^[^ ^136^ ^]^ Copyright 2020, Elsevier.

Most CNM‐based anisotropic foams and aerogels have been prepared by unidirectional ice‐templating, also called freeze‐casting, followed by freeze‐drying. Unidirectional ice‐templating is accomplished by placing the CNM/cellulose dispersion in a cooled mold with the bottom plate that has a much higher heat capacity and thermal conductivity than the walls. This allows the ice to grow unidirectionally and results in anisotropic structures (**Figure** **6**a) as the dispersed CNMs and other particles are expelled from the growing ice crystals. The cooling rate and the concentration of the dispersion can be used, to some extent, to control the pore size and structure.^[^ ^133^, ^134^, ^135^ ^]^

Studies on CNM‐based foams where both the radial and axial thermal conductivities have been determined are sparse. In fact, numerous studies on ice‐templated CNM‐based foams only report one thermal conductivity without specifying if the value represents a specific direction or an average value. In this progress report, we will focus on studies where the experimental results are clearly reported and contain data for both the radial and axial thermal conductivity.

Kriechbaum et al. studied the effect of the degree of nanofibrillation on the thermal conductivity of ice‐templated and FD CNF foams at 295 K and 50% RH using the anisotropic mode of the hot disk.^[^ ^82^, ^87^ ^]^ The results showed that the energy consumption during defibrillation, which controls the degree of nanofibrillation, has a strong effect on the thermal conductivity in both the axial and radial direction (Figure 6b). Increasing the degree of nanofibrillation, i.e., reducing the dimensions of the generated CNF, resulted in a decrease in the radial thermal conductivity and in parallel an increase in the anisotropy of the heat transfer properties. The minimum radial thermal conductivity (18 mW m^−1^ K^−1^) was obtained when the defibrillation and the subsequent ice‐templating resulted in foams with the highest degree of alignment of the columnar macropores (Figure 6c) and of the CNF. Further nanofibrillation resulted in a slightly increased thermal conductivity anisotropy ratio, which could be related to a shortening of the CNF length due to transversal fibrillation, and therefore to an increased number of interfaces in the axial direction. The radial thermal conductivity of the ice‐templated CNF foams studied by Kriechbaum et al.^[^ ^82^ ^]^ was lower than similar materials reported by Gupta et al., where the minimal (radial?) thermal conductivity for a CNF foam of a density of 12 kg m^−3^ was 26 mW m^−1^ K^−1^.^[^ ^86^ ^]^ The difference in radial thermal conductivity of the ice‐templated CNF foams is probably related to differences in the dimensions and degree of alignment of the CNF in the foam walls, and the density of the foams (**Table** **3**). Unfortunately, a lack of information on the dimensions and the degree of alignment makes it difficult to analyze the differences in detail.

**Table 3:** Thermal conductivity of anisotropic CNM/cellulose‐based foams

Refs.	Drying	Cellulose type	Cellulose	Other components	λ_r_ ^c)^/λ_a_ ^d)^	RH^e)^	ρ^f)^	*Π* ^g)^	Pore size
			*d* ^a)^/*L* ^b)^		[mW m^−1^ K^−1^]	[%]	[kg m^−3^]	[%]	
^[^ ^86^ ^]^	FD	CNF^h)^	30 nm/N/A	–	26–39/N/A	0, 65	8–20	98.6–99.4	2–50 nm and µm
^[^ ^82^ ^]^	FD	CNF	N/A/N/A	–	18–22/119–150	50	5.5	99.7	>30 µm
^[^ ^54^ ^]^	FD	TCNF	5 nm/1–2 µm	GO^i)^–BA^j)^–SEP^k)^	15/170	50	7.5	99.5	20 µm and 3 nm
^[^ ^81^ ^]^	FD	TCNF	2.1 nm/N/A	SiO_2_ nanoparticle	21–33/77–132	5–80	19–21	>99	10 µm and 4 nm
^[^ ^136^ ^]^	FD	BC	40–60 nm/N/A	PI^l)^	23/44	N/A	46	97.7	nm to 10 µm
^[^ ^88^ ^]^	FD	Delignified nanowood	N/A/N/A	–	32/56	20	130	91	10–100 µm and nm

a) *d*: diameter

b) *L*: length

c) λ_r_: radial thermal conductivity

d) λ_a_: axial thermal conductivity

e) RH: relative humidity

f) ρ: density

g) *Π*: porosity

h) CNF: cellulose nanofibers

i) GO: graphene oxide

j) BA: boric acid

k) SEP: sepiolite

l) PI: polyimide.

Zhang et al., using 40% BC and 60% polyimide (PI) followed a novel approach to manufacture anisotropic foams (PI/BC) by bidirectional freezing.^[^ ^136^ ^]^ Bidirectional freezing is in principle similar to unidirectional freezing but instead of using a mold with a copper bottom, an inclined polydimethylsiloxane wedge is added on top of the copper in order to create multiple temperature gradients, instead of only one in case of unidirectional freezing (Figure 6c). The multiple temperature gradients led to the formation of distinct and well‐aligned lamellae. The thermal conductivity for the bidirectional PI/BC aerogel was 23 mW m^−1^ K^−1^ along the radial direction and 44 mW m^−1^ K^−1^ along the axial direction, at undefined conditions.

**Figure 7:**
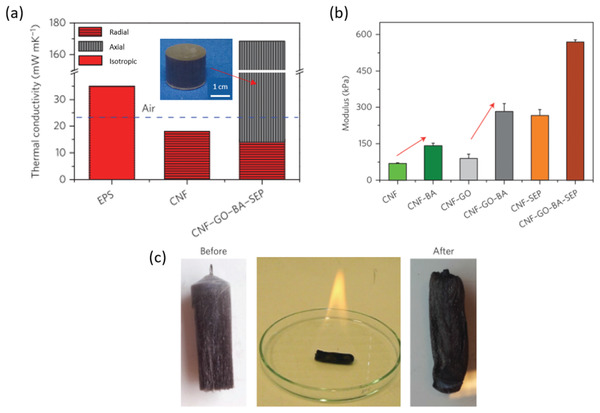
Anisotropic CNF–GO–BA–SEP composite foams and their properties. a) Thermal conductivity of CNF–GO–BA–SEP foams compared to neat CNF foams and expanded polystyrene (EPS) foams. The inset shows a macroscopic view of a CNF–GO–BA–SEP foam. b) Comparison of Young's moduli for neat CNF foams and CNF–GO–BA–SEP foams, as well as for intermediate combinations. c) Illustration of the fire retardancy of a CNF–GO–BA–SEP foam after being soaked in ethanol and burned. Adapted with permission.^[^ ^54^ ^]^ Copyright 2015, The Authors, published by Springer Nature.

The combination of a low radial thermal conductivity, large compressive modulus, and good fire resistance in unidirectional ice‐templated CNM‐based anisotropic TCNF–graphene oxide (GO)–boric acid–sepiolite composite foams (**Figure** **7**) have attracted a large interest.^[^ ^54^ ^]^ The ice‐templated TCNF‐only foams displayed a radial thermal conductivity of 18 mW m^−1^ K^−1^ (Figure 7a) and an axial thermal conductivity of 150 mW m^−1^ K^−1^ (at 295 K and 50% RH), which corresponds well to the values reported by Kriechbaum et al.^[^ ^82^ ^]^ The radial thermal conductivity of the ice‐templated composite foams was as low as 15 mW m^−1^ K^−1^, which is significantly lower than the value for air (Figure 7a). The very low radial thermal conductivity of the composite anisotropic foams was related to the hierarchical structure and the presence of mesopores in the foam walls, which is expected to result in a low gas conduction, due to the Knudsen effect, and to the enhancement of phonon scattering at the interfaces. It is clear that anisotropic foams made by freeze‐casting and freeze‐drying yield tubular macropores with thin and compact walls along the ice growth direction. Anisotropic nanoparticles, e.g., CNM, rod‐like sepiolite clays and flake‐like graphene oxide, will align in the ice‐growth direction as they are forced to assemble in the space between the growing ice crystals. The resulting structure promotes a low radial solid conduction, due to phonon scattering at the nanoscale, and a decreased gas conduction, due to the presence of mesopores in the foam wall, which can result in radial thermal conductivities much lower than air and even comparable to isotropic silica aerogels.^[^ ^19^ ^]^

The anisotropic thermal conductivity of ice‐templated silica nanoparticles–TCNF anisotropic foams was recently investigated from 5% to 80% RH at 295 K,^[^ ^81^ ^]^ using an analogous setup as in Apostolopoulou‐Kalkavoura et al.^[^ ^65^ ^]^ The addition of silica nanoparticles reduced the moisture uptake from 17 wt% for neat TCNF foams to 11 wt% for a TCNF composite with isotropic silica nanoparticles and 16% for a TCNF composite with anisotropic silica nanoparticles (the composition of both composite foams being 67% silica and 33% TCNF). The ice‐templated TCNF‐only foams displayed a radial thermal conductivity below the superinsulating level for 20–80% RH with a minimum value of 18 mW m^−1^ K^−1^ at 65% RH. The radial thermal conductivity of the silica nanoparticles–TCNF foams, with a 3 times higher density compared to the neat TCNF foams, remained below the superinsulating level for 0–35% RH, with a minimum value of 21 mW m^−1^ K^−1^ at 5% RH. The very low radial thermal conductivity of the silica nanoparticles–TCNF composite foams was related to a reduction of the gas conduction due to a significant increase in the mesoporosity of the foam walls with the addition of silica, from only 4% mesoporosity for neat TCNF to 16% for the TCNF composite with isotropic silica nanoparticles and 23% for the TCNF composite with anisotropic silica nanoparticles.^[^ ^81^ ^]^ The average pore size in the foam walls also decreased from 10 nm for the ice‐templated TCNF‐only foams to around 4 nm for the silica nanoparticles–TCNF composite foams, which is also expected to reduce the gas conduction due to the Knudsen effect. It is interesting to note that the radial thermal conductivities of ice‐templated CNF foams at RH < 5% were reported between 28 and 30 mW m^−1^ K^−1^,^[^ ^81^, ^86^ ^]^ while the values at 50% RH were substantially lower and ranged between 18 and 20 mW m^−1^ K^−1^.^[^ ^54^, ^81^, ^82^ ^]^ This suggests that the moisture uptake can have a significant effect on the thermal conductivity of anisotropic foams and that there is a need to investigate the RH‐dependence of the thermal conductivity of CNM‐based foams in detail.

The unidirectional freezing and freeze‐drying of CNF aerogels has also been combined with various post‐treatments. Coating the walls of ice‐templated CNF foam with carbon nanotubes (CNTs) for solar steam generation, resulted in a thermal conductivity of 60 mW m^−1^ K^−1^ (probably radially but unspecified) at undefined *T* and RH.^[^ ^137^ ^]^ This relatively high thermal conductivity is probably due to the addition of 26 wt% of randomly distributed CNT, which displays a thermal conductivity of 2–6 × 10^6^ mW m^−1^ K^−1^.^[^ ^138^ ^]^ Nanowood, i.e., delignified wood, is a cellulosic material that preserves the intrinsic structure and fibrillar alignment in wood.^[^ ^88^ ^]^ The removal of the lignin and hemicelluloses, which are mainly interconnecting the fibril aggregates, results in an increase in porosity, including the mesoporosity, and a decrease in density. Consequently, the radial thermal conductivity (perpendicularly to the lumen/fiber direction) dropped from 107 mW m^−1^ K^−1^ (at 298 K and 20% RH) for American basswood (Tilia Americana) to 32 mW m^−1^ K^−1^ for the corresponding nanowood, and the axial thermal conductivity (along the lumen/fibers direction) dropped from 347 mW m^−1^ K^−1^ for basswood to 56 mW m^−1^ K^−1^ for nanowood. Nanowood is hygroscopic and an increase in RH from 20 to 80% at 298 K resulted in an increase in the thermal conductivities in the radial and axial directions to 55 and 100 mW m^−1^ K^−1^, respectively.

## 6 Anisotropic CNM‐Based Films

**Figure 8:**
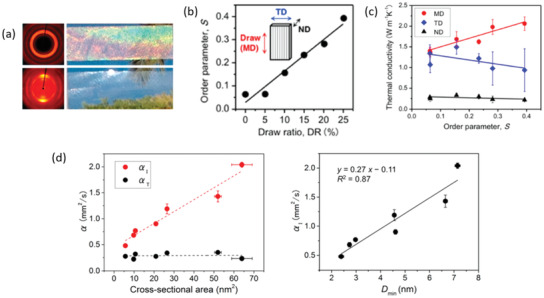
Anisotropic thermal conductivity of oriented CNM films. a) Macroscopic views of disordered (top) and shear‐oriented (bottom) CNC films, with the corresponding 2D‐X‐ray diffraction (XRD) patterns. b) Graphical illustration of the increase in the order parameter with increasing mechanical drawing ratios for bacterial cellulose nanopapers. The inset shows the three characteristic directions of the nanopapers, namely, the machine direction (MD), the transverse direction (TD), and the normal direction (ND). c) Evolution of the thermal conductivity as a function of the order parameter in the three characteristic directions of bacterial cellulose nanopapers. d) Left: evolution of the in‐plane (α_I_) and out‐of‐plane (α_T_) thermal diffusivities in nanocellulose films as a function of the cross‐sectional area of the particles. Right: α_I_ as a function of minimum crystallite size (*D*_min_). a) Adapted with permission.^[^ ^48^ ^]^ Copyright 2014, American Chemical Society. b,c) Adapted with permission.^[^ ^140^ ^]^ Copyright 2017, American Chemical Society. d) Adapted with permission.^[^ ^105^ ^]^ Copyright 2015, American Chemical Society.

Films and substrates with anisotropic heat transfer properties are already used for the thermal management of, e.g., electronic devices where heat needs to be transported to heat sinks and not only dissipated. Films with highly aligned CNMs have exploited the intrinsic anisotropy of cellulose molecules and the phonon scattering at the nanoscale to generate materials with anisotropic heat transport properties. CNC films that have been oriented by shear fields^[^ ^48^ ^]^ displayed order parameters, *S*, up to *S* ≈ 0.76 and an in‐plane thermal conductivity at RT and vacuum conditions (530 mW m^−1^ K^−1^) that was twice as high compared to nonaligned CNC films (**Figure** **8**a). The out‐of‐plane radial thermal conductivity decreased for *S* > 0.63 as phonon scattering became more dominant when the number of interfaces increased along the heat flow direction. The addition of CNF to epoxy resin films^[^ ^139^ ^]^ was shown to increase the anisotropy ratio from 0, for the epoxy resin‐only film, to 4.8, for the vacuum‐filtrated CNF–epoxy resin nanocomposite films, with thermal conductivities of 1100 and 230 mW m^−1^ K^−1^ in the in‐plane and out‐of‐plane directions, respectively.

Mechanical drawing was used to prepare a film, or so called nanopaper, based on aligned BC.^[^ ^140^ ^]^ The order parameter, that was controlled by the mechanical drawing conditions (Figure 8b), was related to the anisotropic thermal conductivity (Figure 8c). The thermal conductivity became more anisotropic as the order parameter increased, reaching a maximal anisotropy ratio (2.23) at *S* = 0.4, with thermal conductivities of 2100 and 940 mW m^−1^ K^−1^ (undefined *T* and RH) in the drawing and transversal directions, respectively. In comparison, the thermal conductivity of the isotropic films was 1300 mW m^−1^ K^−1^. Extrapolation suggested that the heat transport anisotropy ratio at ideal orientation (*S* = 1) would be 9 and the in‐plane thermal conductivity would be as high as 3400 mW m^−1^ K^−1^.

The dimensions and degree of crystallinity of different types of CNM can also influence the heat transport properties of anisotropic CNM films.^[^ ^105^ ^]^ Oriented CNM films prepared by filtration and hot pressing using a variety of CNMs, e.g., tunicate nanowhiskers, cotton nanowhiskers, sugi TCNF, and sugi CNF were used to compare the effect of crystallite size on the thermal conductivity (Figure 8d). CNMs with larger crystallite sizes, determined by X‐ray diffraction (XRD), induced higher anisotropy ratios. Films made from tunicate nanowiskers with the largest crystallite size (>7 nm) exhibited thermal conductivities of 2470 and 290 mW m^−1^ K^−1^ in the in‐plane and out‐of‐plane directions, respectively (at undefined *T* and RH conditions), achieving an anisotropy ratio of 8.5.

There have also been some attempts to increase the anisotropy ratio even more while maintaining a relatively low out‐of‐plane thermal conductivity, by addition of other nanomaterials with highly anisotropic heat transfer properties, e.g., GO. CNF–GO hybrid films, prepared by filtration and layer‐by‐layer dip coating of alternate CNF and GO layers, displayed an in‐plane thermal conductivity as high as 12 600 mW m^−1^ K^−1^ and an out‐of‐plane thermal conductivity as low as 42 mW m^−1^ K^−1^ (at 298 K but undefined RH), resulting in an anisotropy ratio of 279 for a hybrid of 40 layers.^[^ ^141^ ^]^ However, the corresponding CNF‐only films exhibited a thermal conductivity of 1100 mW m^−1^ K^−1^ in the in‐plane direction and only 34 mW m^−1^ K^−1^ in the out‐of‐plane direction. It should be noted that the reported out‐of‐plane thermal conductivity is almost an order of magnitude lower than any other reports on CNF films. CNF–GO composite films have also been prepared by vacuum filtration of CNF–GO suspensions.^[^ ^89^ ^]^ For nanosheets containing 30% GO, the thermal conductivity was 6168 and 72 mW m^−1^ K^−1^ in the in‐plane and out‐of‐plane directions, respectively (at 298 K but undefined RH), thus yielding an anisotropy ratio of 86. Composite films of CNF and reduced graphene oxide (RGO) resulted in an anisotropy ratio of 56 at an RGO content of 50%, where the in‐plane thermal conductivity was 7300 mW m^−1^ K^−1^ and the out‐of‐plane thermal conductivity was 130 mW m^−1^ K^−1^ at ambient conditions.^[^ ^142^ ^]^

As an alternative to GO, Wang and Wu prepared CNF‐based films containing 35% fluorinated CNT by vacuum filtration.^[^ ^108^ ^]^ The CNF‐fluorinated CNT films displayed an in‐plane thermal conductivity of 14 100 mW m^−1^ K^−1^ and an out‐of‐plane thermal conductivity of 830 mW m^−1^ K^−1^ (undefined *T* and RH), corresponding to an anisotropy ratio of 17. Comparing the addition of GO and CNT to generate films with anisotropic thermal conductivities suggests that GO induces a substantially larger anisotropy compared to CNT; this could be due to the sheet/platelet shape of GO particles as opposed to the 1D CNT, which therefore have a lesser amount of out‐of‐plane weak bonds.

## 7 Conclusions and Outlook

**Figure 9:**
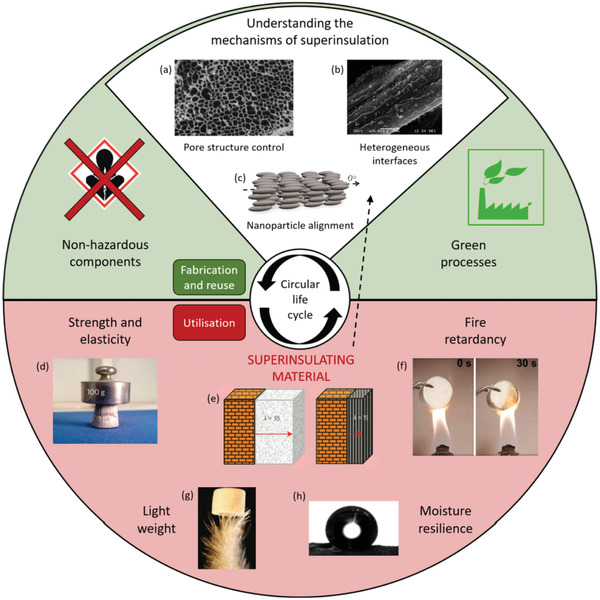
Requirements for cellulose nanomaterial‐based insulation materials. a–h) Schematic summarizing the requirements and fundamental challenges for sustainable CNM‐based insulation materials related to the performance and circular life cycle. a) Adapted with permission.^[^ ^133^ ^]^ Copyright 2016, American Chemical Society. b) Adapted with permission.^[^ ^120^ ^]^ Copyright 2015, Elsevier. c) Adapted with permission.^[^ ^48^ ^]^ Copyright 2014, American Chemical Society. d,e) Adapted with permission.^[^ ^54^ ^]^ Copyright 2015, The Authors, published by Springer Nature. f,g) Adapted under the terms and conditions of the CC BY 4.0 license.^[^ ^83^ ^]^ Copyright 2020, The Authors, published by Springer Nature. h) Adapted with permission.^[^ ^143^ ^]^ Copyright 2013, American Chemical Society.

In this progress report, we have described the fundamental aspects of heat transfer and thermal conductivity of cellulose nanomaterials, and provided a detailed account of the seminal publications describing CNM‐based aerogels and foams with low thermal conductivities. During the last 5 years, several examples of superinsulating, light‐weight, and strong CNM‐based aerogels and foams have been reported, which shows that there is a large potential to use CNMs to produce renewable thermally insulating materials with a significantly better heat transport properties than the commercially dominating materials such as expanded polystyrene, polyurethane foams, and glass wool (**Figure** **9**).

Analysis of the different contributions to the thermal conductivity of porous materials shows that the gas and solid conduction contributions dominate. The gas conduction contribution can be significantly reduced if the pore size is smaller than the mean free path of air molecules, and several studies on, e.g., supercritically dried aerogels have shown that a decrease in pore size below 50 nm results in a decrease in thermal conductivity due to an enhancement of the Knudsen effect. However, a detailed analysis of the relation between the pore structure and heat transfer properties is difficult to perform because the pore size distributions of CNM‐based aerogels and foams are usually quite wide and the pore connectivity, which may be important for convection effects, is often poorly defined.

The solid contribution to the thermal conductivity can be reduced by phonon scattering at interfaces, which suggests that the density and composition of solid–solid and solid–gas interfaces are important to control and engineer to reduce the thermal conductivity of CNM‐based materials. There are indications that the dimensions (i.e., diameter and length) and surface modification of the fibrils can have a significant impact on phonon scattering, with thin fibrils resulting in foams or aerogels with numerous interfaces and a low thermal conductivity. However, there is a need for more in‐depth studies that systematically investigate how the heat transport depends on the structural features of both the CNMs and the aerogels and foams.

CNM‐based foams and films with anisotropic structures and aligned CNMs can display highly anisotropic heat transfer properties that could be of interest in thermal management of devices, food products, pharmaceuticals, and the environment in buildings. Films with highly aligned tunicate nanowhiskers displayed a heat transport anisotropy of 8.5, which is similar to the intrinsic anisotropic of CNC. Ice‐templated foams with an anisotropic pore structure and aligned fibrils can exhibit superinsulating thermal conductivities perpendicular to the fibers, which suggests that the interfacial thermal resistance in this direction is large.

It is well known that CNMs are usually hygroscopic but there are a very limited number of studies that have investigated how moisture uptake modulates heat transport and thermal conductivity of CNM‐based foams and aerogels. In fact, there is a general need to improve the procedure and reporting of thermal conductivity data and specify the temperature and relative humidity for the measurements.

CNM‐based thermally insulating materials could contribute to the transition from a linear to a circular economy (Figure 9) that reduces the use of fossil resources through green processes and nonhazardous compounds and additives. The extraordinarily low thermal conductivity of some CNM/cellulose‐based aerogels and foams could reduce the energy consumption related to heating and cooling in new buildings or retrofitted old buildings. The scale of the building industry calls for the development of scalable and energy‐efficient processes to produce isotropic as well as anisotropic CNM‐based aerogels and foams. SCD and FD yield materials with tunable structures but the methods are slow and energy intensive. There are promising examples of how the capillary stresses during oven or ambient drying can be mitigated by crosslinking, hydrophobization, or reinforcement^[^ ^65^, ^73^, ^74^, ^132^ ^]^ but more research on scalable production methods of anisotropic foams is needed.

Thermally insulating materials also need to be strong and lightweight with good fire‐retardant properties. The mechanical strength can be increased by crosslinking^[^ ^54^, ^84^, ^131^ ^]^ and the fire retardancy of CNM‐based materials has been improved by the addition of clays and other inorganic nanomaterials.^[^ ^54^, ^59^, ^83^, ^84^ ^]^ The demonstration that ice‐templated CNF‐based foams with addition of graphene oxide, boric acid, and sepiolite could enhance the mechanical properties and the fire retardancy, while retaining a radial thermal conductivity as low as 15 mW m^−1^ K^−1^,^[^ ^54^ ^]^ is very promising and inspiring for further studies of CNM‐based hybrids and nanocomposites.

## Conflict of Interest

The authors declare no conflict of interest.
